# Protoporphyrin IX enhancement by 5-aminolaevulinic acid peptide derivatives and the effect of RNA silencing on intracellular metabolism

**DOI:** 10.1038/sj.bjc.6604928

**Published:** 2009-02-24

**Authors:** L Bourré, F Giuntini, I M Eggleston, M Wilson, A J MacRobert

**Affiliations:** 1 Division of Surgical and Interventional Sciences, National Medical Laser Centre, UCL Medical School, University College London, Charles Bell House, 67–73 Riding House St, London W1W 7EJ, UK; 2 Department of Pharmacy and Pharmacology, University of Bath, Claverton Down, Bath BA2 7AY, UK; 3 Division of Microbial Diseases, UCL Eastman Dental Institute, University College London, 256 Grays Inn Road, London WC1X 8LD, UK

**Keywords:** photodynamic therapy, aminolaevulinic acid, ALA derivatives, acylpeptide hydrolase (ACPH, EC 3.4.19.1), siRNA silencing, protoporphyrin IX

## Abstract

Intracellular generation of the photosensitiser, protoporphyrin IX, from a series of dipeptide derivatives of the haem precursor, 5-aminolaevulinic acid (ALA), was investigated in transformed PAM212 murine keratinocytes, together with studies of their intracellular metabolism. Porphyrin production was substantially increased compared with equimolar ALA using *N*-acetyl terminated phenylalanyl, leucinyl and methionyl ALA methyl ester derivatives in the following order: Ac-L-phenylalanyl-ALA-Me, Ac-L-methionyl-ALA-Me and Ac-L-leucinyl-ALA-Me. The enhanced porphyrin production was in good correlation with improved photocytotoxicity, with no intrinsic dark toxicity apparent. However, phenylalanyl derivatives without the acetyl/acyl group at the N terminus induced significantly less porphyrin, and the replacement of the acetyl group by a benzyloxycarbonyl group resulted in no porphyrin production. Porphyrin production was reduced in the presence of class-specific protease inhibitors, namely serine protease inhibitors. Using siRNA knockdown of acylpeptide hydrolase (ACPH) protein expression, we showed the involvement of ACPH, a member of the prolyl oligopeptidase family of serine peptidases, in the hydrolytic cleavage of ALA from the peptide derivatives. In conclusion, ALA peptide derivatives are capable of delivering ALA efficiently to cells and enhancing porphyrin synthesis and photocytotoxicity; however, the N-terminus state, whether free or substituted, plays an important role in determining the biological efficacy of ALA peptide derivatives.

Photodynamic therapy (PDT) is a promising modality for cancer treatment that utilises tumour-specific accumulation of a photosensitiser, followed by irradiation with light at an appropriate wavelength (Bonnett, 1995). The use of 5-aminolaevulinic acid (ALA) for PDT has received considerable interest owing to the metabolism of ALA through the haem biosynthetic pathway (Peng *et al*, 1997) to the photosensitiser protoporphyrin IX (PpIX). Following the exogenous administration of ALA, PpIX is generated intracellularly, which can then be activated by visible light for PDT treatment. ALA-PDT does exhibit a degree of intrinsic tumour selectivity owing to differing tumour levels of enzymes in the haem pathway (e.g. ferrochelatase) leading to higher tumour PpIX levels. The principal advantages of ALA-PDT are the short duration of skin photosensitivity and its efficacy using both topical and oral administration (Peng *et al*, 1997; Brown *et al*, 2004).

A significant drawback of ALA-PDT is the fact that ALA is a zwitterion at physiological pH resulting in low lipid solubility and limiting passage through biological barriers such as cellular membranes. To overcome this problem, several chemical approaches have been attempted to improve the incorporation of ALA and also its selectivity. One approach has been to use more lipophilic ALA derivatives, such as alkyl or ethyleneglycol esters, which are potential substrates for cellular esterases (Kloek and Beijersbergen, 1996; Gaullier *et al*, 1997; Berger *et al*, 2000; Uehlinger *et al*, 2000), or different delivery systems including dendrimers (Battah *et al*, 2001, 2006; Di Venosa *et al*, 2006), or liposomes (Casas *et al*, 2002; Casas and Batlle, 2006). 5-Aminolaevulinic acid prodrug ester derivatives have been widely studied (Kloek and Beijersbergen, 1996; Peng *et al*, 1996; Gaullier *et al*, 1997; Kloek *et al*, 1998; Casas *et al*, 1999; Eleouet *et al*, 2000; Brunner *et al*, 2003; Lopez *et al*, 2004), in particular the methyl and hexyl ester derivatives, and approval has been granted for the treatment of actinic keratosis and basal cell carcinoma using the methyl ester derivative in Europe and Australia. The use of alkyl esters of ALA results in a nonspecific distribution of ALA in all cell types, but with an increased PpIX production (Webber *et al*, 1997; Uehlinger *et al*, 2000) in tumour cells. In this regard, Casas *et al* (1999) and Berger *et al* (2000, 2003) have conjectured that the incorporation of 5-ALA into a short peptide derivative would provide a suitable means of both facilitating transdermal delivery and also improved targeting to cancerous cells.

In an attempt to improve uptake and cell selectivity of ALA and its derivatives, we investigated PpIX formation and metabolism mechanisms using peptide ALA prodrugs that represent potential substrates for cell-surface and cytoplasmic peptidases and esterases. A recent investigation of ALA prodrugs showed that the L-enantiomer of Ac-Phe-ALA-Me, **1**, was capable of delivering ALA to cells, with an enhancement of PpIX and PDT effectiveness, comparable with the results obtained with the hexyl ester derivative, ALA-Hex (Bourré *et al*, 2008). The ALA residue was masked on one side through a peptide bond and on the other side through an ester linkage, which were then cleaved enzymatically within the cells to liberate the ALA. We also showed that the D-enantiomer did not induce any PpIX production, which would be consistent with the stereospecific enzymatic cleavage acting only on the L-forms of such prodrugs.

In this study, we have focused on several ALA peptide derivatives using different amino acids and have studied the influence of structurural modification at the N terminus by changing the N-terminal blocking group. We then studied the metabolism mechanism involved in the release of ALA using class protease inhibitors and silencing of mammalian acylpeptide hydrolase activity (ACPH, EC 3.4.19.1) using small interfering RNA (siRNA) technology. Acylpeptide hydrolase, a member of the prolyl oligopeptidase family of serine peptidases, is an exopeptidase that removes acylated amino-acid residues from the N terminus of oligopeptides.

## Materials and methods

### Chemicals

5-Aminolaevulinic acid was purchased from Sigma-Aldrich Ltd (Gillingham, UK).

**Figure 1:**
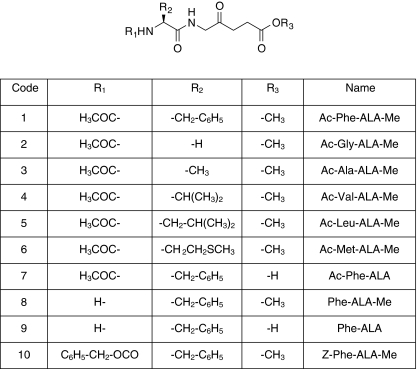
General structure of ALA peptide derivatives.

The structures of ALA peptide derivatives are shown in Figure 1. The synthesis of compounds **1**–**5** (Rogers *et al*, 2005) and **8** (Bourré *et al*, 2008) has already been reported. Compounds **6**, **9** and **10** were synthesised according to the same procedure with minor modifications. Stock solutions of compounds **1** and **2** were prepared by dissolving at 10 mM in 0.01 M HCl and stored at −20°C. Compound **3** was dissolved at 100 mM in 0.1 M HCl solution and stored at −20°C.

### Cell culture

The spontaneously transformed murine keratinocyte cell line, PAM212 (obtained from Professor R Groves, King's College, London), was cultured in RPMI-1640 medium (Gibco BRL, Life Technologies Ltd, Paisley, UK) containing L-glutamine (2 mM) and phenol red, supplemented with 10% foetal calf serum (FCS; Sigma-Aldrich Ltd) and penicillin and streptomycin (500 U ml^−1^ and 0.5 mg ml^−1^; Gibco BRL). The cells were routinely grown as monolayers in 75-cm^2^ culture flasks (TPP; Helena Bioscience, Gateshead, UK) at 37°C, 5% CO_2_ atmosphere until confluent.

### Porphyrin synthesis determination

Cells were seeded into *γ*-sterilised 96-well plates (Orange Scientific, Triple Red Laboratory Technologies, Long Crendon, UK) at a density of 5 × 10^4^ cells per well for 48 h. After removing the culture medium, the wells were washed with PBS and incubated with freshly prepared solutions of ALA or ALA peptide derivatives **1**–**10**: 0.1 ml of serum-free medium containing varying prodrug concentrations was added to a designated series of wells. Each plate contained control wells with cells but without added drug for the determination of the background reading and reference wells containing cells incubated with the same ALA concentrations. For drug incubation, serum-free medium was used as serum is known to cause the release of PpIX from cells, thus resulting in the loss of fluorescence signal (Kloek *et al*, 1998).

The fluorescence signal from each well was measured with a spectrometer Perkin-Elmer LS 50B fluorescence spectrometer (Perkin Elmer, Beaconsfield, UK) using 410-nm excitation and 635-nm emission wavelengths, with slit widths set to 10 nm and the internal 530 nm longpass filter used on the emission side; spectral scans were recorded between 600 and 750 nm to check for the presence of any porphyrins other than PpIX (Battah *et al*, 2006). The mean fluorescence intensity (expressed in arbitrary units) was calculated after subtraction of the control values. Intensity calibrations were performed using rhodamine B embedded in a Perspex disc as a standard.

### Photodynamic treatment

Cells were seeded into 96-well plates at a density of approximately 2 × 10^4^ cells per well. Following incubation for 48 h, the cells were washed with PBS, and 0.1 ml of solutions containing ALA, 1, 6, 7, 8 and 9 at 0.01 mM were added to their designated wells for 4-h incubation periods. The plates were irradiated with a fluence of 2 J cm^−2^ using a LumiSource lamp (PCI Biotech, Oslo, Norway) that emits a uniform field of low-power blue light over an area of 14 × 32 cm. Peak output is around 420 nm, which overlaps well with the PpIX Soret band. Immediately following irradiation, the medium was replaced and cells were incubated for a further 24 h. Cell cytotoxicity was determined using the 3-(4,5-dimethylthiazol-2-yl)-2,5-diphenyltetrazolium bromide (MTT) assay: cells were incubated with medium containing MTT (1 mg ml^−1^ dissolved in full RPMI-1640 medium) for 3 h. The insoluble end product (formazan derivatives) was dissolved in 0.1 ml of dimethylsulphoxide after removing the medium. Ultraviolet absorption was quantified at 570 nm using a 96-well plate reader (MR 700 Dynatech; Dynex, Worthing, UK). The mean cell survival was calculated for each prodrug at every concentration tested and expressed as a percentage of control (incubated with the compounds but not irradiated) cell survival values. For the determination of ‘dark’ toxicity of the compounds, well plates were prepared in the same manner as above but without irradiation.

### Peptidase activities

#### Influence of protease inhibitors

Evaluation of PpIX production by fluorescence recording in living cells, performed as described earlier above, was determined after incubation with a range of protease inhibitors. The protease inhibitors were added to cell cultures 10 min before adding ALA or compound **1** at 0.1 mM and fluorescence increases were recorded. Protease inhibitors used are protease inhibitor cocktail (1 : 250), aprotinin (competitive serine protease inhibitor) (20 *μ*M), AEBSF (irreversible serine protease inhibitor) (200 *μ*M), 2,2′-bipyridyl (metalloprotease inhibitor) (20 *μ*M), bestatin (metalloaminopeptidase inhibitor) (100 *μ*M), E64 (cysteine protease inhibitor) (10 *μ*M), leupeptin (trypsin-like protease and cysteine protease inhibitor) (100 *μ*M) and captopril (angiotensin-converting enzyme inhibitor) (20 *μ*M). Protease inhibitors were purchased from Sigma-Aldrich Ltd.

#### Acylpeptide hydrolase assay

PAM212 cells were solubilised with CelLytic and centrifuged at 13 000 g for 5 min. The supernatant (cell lysate) was assayed for ACPH activity using 4 mM acetyl-alanine *p*-nitroanilide (AcApNA) (Kobayashi and Smith, 1987; Scaloni *et al*, 1992b; Yamaguchi *et al*, 1999) as substrate in 20 mM Tris-HCl (pH 7.4) (Scaloni *et al*, 1994). The influence of thiol reagent pHMB and histidine-modifying reagent DEP on ACPH activity was also assessed. The solution was kept at 37°C and the liberated *p*-nitroaniline from AcApNA was measured at 405 nm using a 96-well plate reader (MR 700 Dynatech) at 0, 10, 30, 60 and 120 min.

The influence of ALA, Ac-Ala-Ala-Ala-Ala-OH and **1** on ACPH activity was evaluated as described above except that the cell lysate was preincubated with these compounds for 1 h.

### Knockdown of ACPH protein expression with siRNA

For each well transfected in 12-well plates, 60 pmol siRNA (ACPH siRNA; Invitrogen, Paisley, UK) was diluted in 200 *μ*l of serum-free Opti-MEM I Medium (Invitrogen). Then 2 *μ*l of Lipofectamine RNAiMAX was added to each well containing the diluted siRNA molecule and incubated for 20 min at room temperature.

PAM212 cells (3 × 10^4^ per ml in RPMI with serum, without antibiotics) were seeded in 12-well plates (1 ml per well) and allowed to grow for 72 h, 37°C, 5% CO_2_, before experimentation. The PpIX synthesis was assessed after incubation with 0.5 mM of ALA or **1** during 4 h, and then washed with PBS. One ml of CelLytic (Sigma-Aldrich Ltd) was added and incubated during 15 min at room temperature, and then the cells were mechanically scraped. Cell extracts were centrifuged at 1800 g for 10 min to remove the cell debris, and the supernatant containing the PpIX was collected. The fluorescence of extracted PpIX was measured as described earlier.

### Statistical analysis

The results are displayed graphically, with error bars representing standard deviations. Differences are considered to be significant using the unpaired *t*-test.

## Results

### PpIX synthesis determination

**Figure 2:**
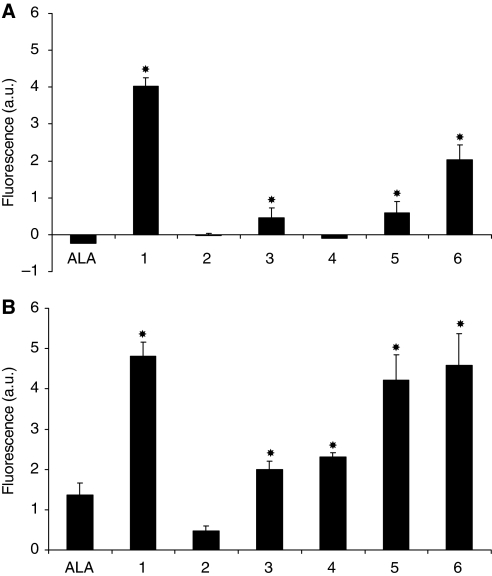
PpIX fluorescence in PAM212 cells after incubation at 37°C for 4 h with 0.01 mM (**A**) or 0.1 mM (**B**) of ALA peptide derivatives **1**–**6** and ALA. Error bars show standard deviations. ^*^P<0.01 (ALA *vs* other compounds).

The PpIX produced by exposure to compounds **1–6**, compared with ALA, is shown in Figure 2A and B. At 0.01 mM (Figure 2A), ALA was not able to produce any measurable PpIX; however, a high amount of PpIX was produced with **1**. Compounds **3**, **5** and **6** also induced some PpIX production but to a lesser extent. However, compounds **2** and **4** were not able to induce measurable PpIX levels at this concentration. Using 0.1 mM (Figure 2B), the level of PpIX was enhanced by a factor 3.5 with **1**, **5** and **6** compared with ALA. Compounds **3** and **4** enhanced the PpIX production by a factor 1.5; however, **2** produced less PpIX compared with ALA.

### Fluorescence pharmacokinetics

**Figure 3:**
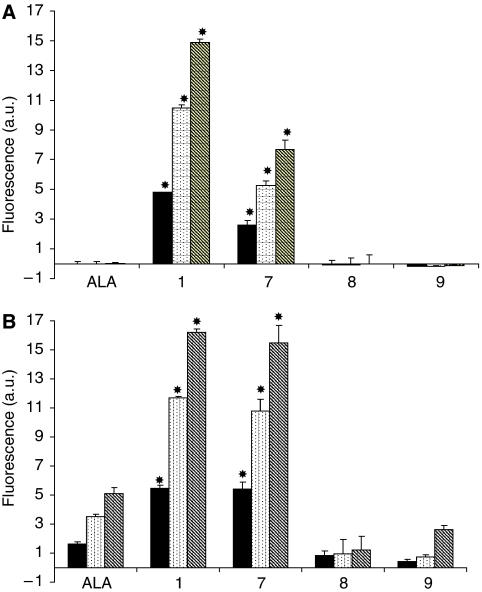
PpIX fluorescence in PAM212 cells exposed at 37°C for 2 (▪), 4 (
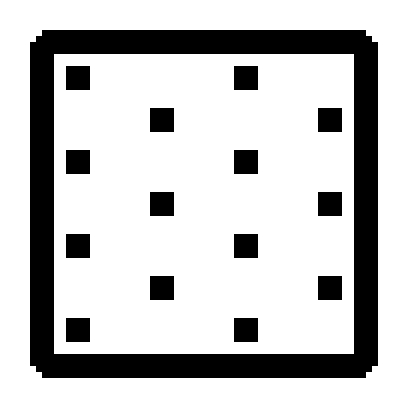
) and 6 h (
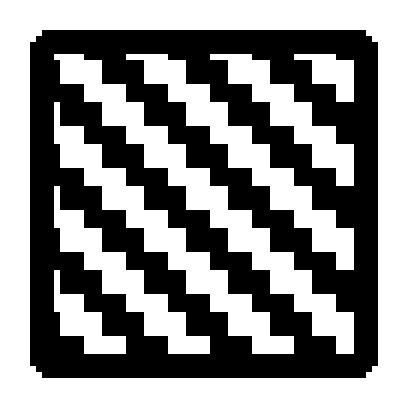
) with 0.01 mM (**A**) or 0.1 mM (**B**) of ALA, **1**, **7**–**9**. Error bars show standard deviations. ^*^P<0.01 (ALA *vs* other compounds).

The kinetics of intracellular porphyrin fluorescence induced by exposure to compounds **1**, **7**–**9** and ALA in the PAM212 cell line are shown in Figure 3A and B. Fluorescence spectral profiles were consistent with the production of PpIX, and peak emission at 635 nm was used for the pharmacokinetic measurements. Increasing porphyrin levels were observed with increasing incubation times from 2 to 6 h. For the lowest concentration tested, 0.01 mM, no PpIX production was observed with ALA, **8** and **9**. However, **1** and **7** induced high levels of PpIX, **1** inducing the higher level regardless of the incubation time. When the concentration was increased to 0.1 mM, all the compounds induced PpIX production, with a significant increase found with **1** and **7**. However, peptides **8** and **9**, compounds without an acetyl group at the N terminus, induced less PpIX compared with ALA; these results were also observed in human epidermoid carcinoma A431 cells and colon carcinoma Caco-2 cells (data not shown). No significant difference was observed for **1** when the concentration was increased.

**Figure 4:**
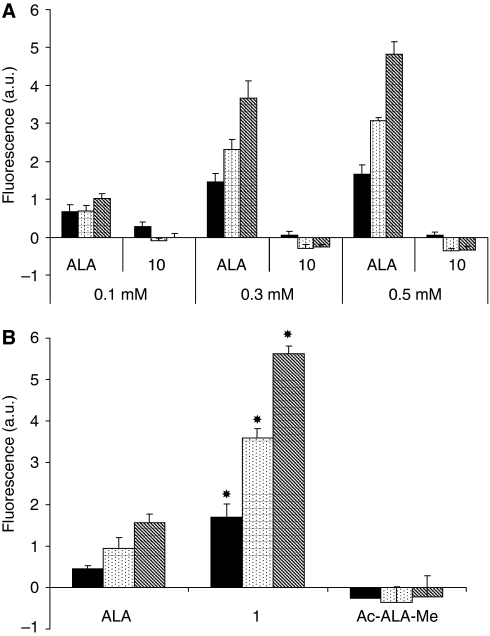
(**A**) PpIX fluorescence in PAM212 cells exposed at 37°C for 2 (▪), 4 (
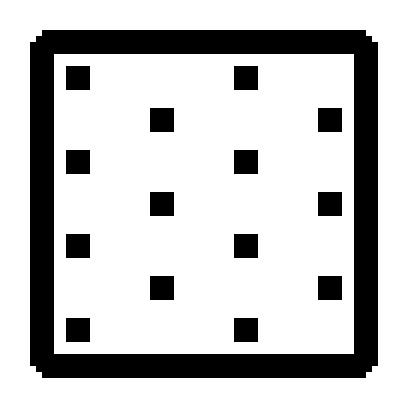
) and 6 h (
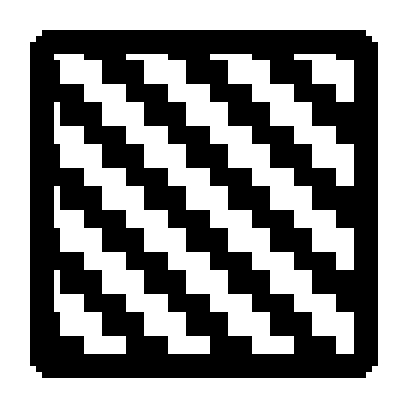
) with 0.1, 0.3 and 0.5 mM of ALA or **10**. Error bars show standard deviations. ^*^P<0.01 (ALA *vs* **10**). (**B**) PpIX fluorescence in PAM212 cells exposed at 37°C for 2 (▪), 4 (
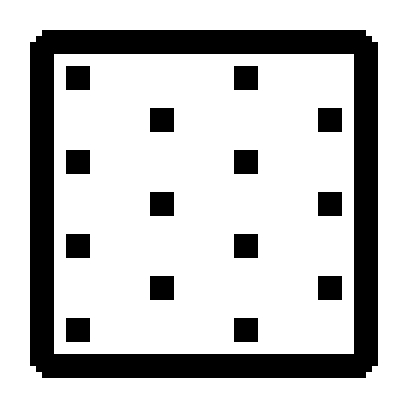
) and 6 h (
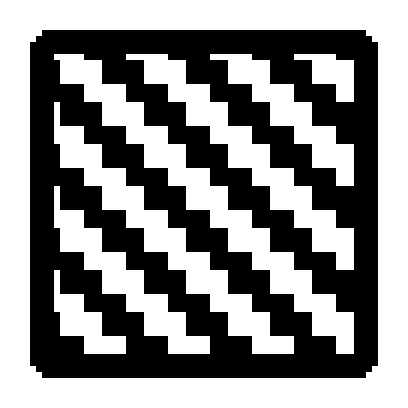
) with 0.1 mM of ALA, **1**, Ac-ALA-Me. Error bars show standard deviations. ^*^P<0.01 (ALA *vs* other compounds).

Figure 4A shows the results of experiments carried out with compound **10** *vs* **1** and ALA, to observe the effect of the Z group at the N terminus instead of the acetyl group in compound **1**. No PpIX production was observed with **10** compared with ALA, whatever the incubation time or the concentration used. In Figure 4B, the effect of the presence of the amino acid in the prodrug derivative was studied, and for this purpose, the molecule, Ac-ALA-Me, was synthesised. No PpIX production was observed with Ac-ALA-Me compared with ALA or **1**, whatever the incubation time or the concentration used.

### Photodynamic treatment

**Figure 5:**
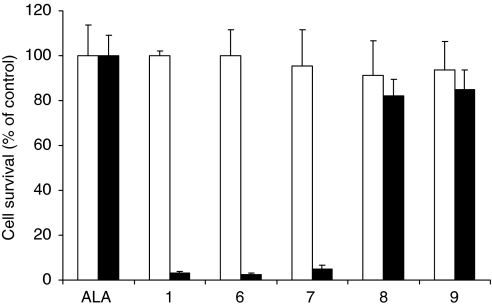
Toxicity (□) and phototoxicity (▪) after incubation with ALA, **1**, **6**, **7**, **8** and **9** at 0.01 mM in PAM212 cell lines assessed by the MTT assay: cells were incubated with the compounds for 4 h and irradiated with blue light (2 J cm^−2^). Error bars show standard deviations.

The phototoxicity was investigated after 4 h of incubation with 0.01 mM of ALA, **1**, **6**, **7**, **8** and **9**. The percentage of cell survival with respect to control cells (without compounds) was calculated and plotted as shown in Figure 5. No dark toxicity was found for any of the compounds. Compounds **1**, **6** and **7** exhibited a high phototoxicity with less than 5% cell survival. However, no phototoxicity was observed with ALA, **8** and **9**, with 100, 82 and 84% of cell survival, respectively.

### Peptidase activities: acylpeptide hydrolase assay

#### Influence of protease inhibitors

**Figure 6:**
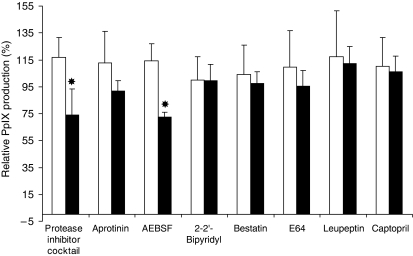
Relative PpIX production (%) in PAM212 cells exposed to class-specific protease inhibitors after 4 h of incubation with 0.1 mM of ALA (□) or **1** (▪) at 37°C. Percentage was expressed compared with the cells treated with ALA or **1** without protease inhibitors. Error bars show standard deviations. ^*^P<0.05.

To obtain information concerning the enzymatic activities involved in releasing free ALA from these precursors, we evaluated PpIX production after incubation with ALA or compound **1** in the presence of class-specific protease inhibitors (Figure 6): protease inhibitor cocktail, aprotinin, AEBSF, 2,2′-bipyridyl, bestatin, E64, leupeptin and captopril.

Aprotinin, bestatin, 2,2′-bipyridyl, E64, leupeptin and captopril had no significant effect. However, the PpIX production was reduced by serine protease inhibitor AEBSF.

#### Acylpeptide hydrolase assay

**Figure 7:**
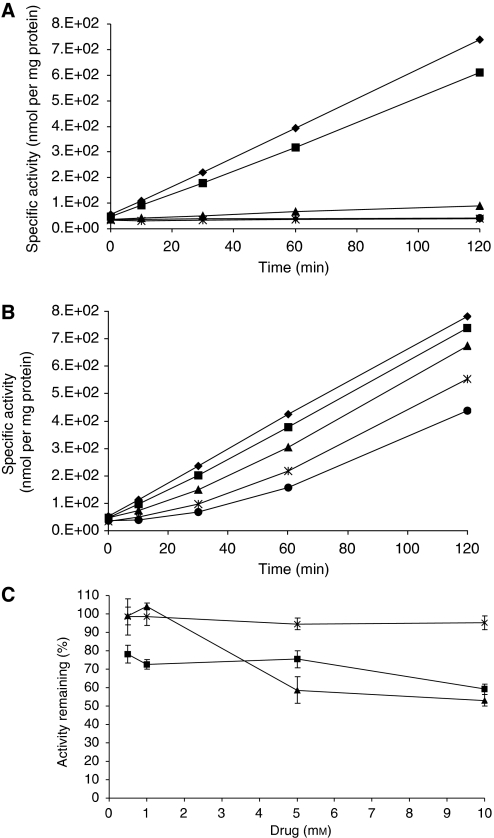
Inactivation of acylpeptide hydrolase by thiol reagent pHMB (**A**) and histidine-modifying reagent DEP (**B**). Cellular extracts were incubated with 0 mM (⧫), 0.01 mM (▪), 0.05 mM (▴), 0.2 mM (*) and 0.5 mM (•) of pHMB or DEP. (**C**) Influence of ALA (*), Ac-Ala-Ala-Ala-Ala-OH (▴) and **1** (▪) on acylpeptide hydrolase activity. Cellular extracts were preincubated with a range of concentration of prodrugs during 1 h, then the enzyme activity was determined with AANA as substrate as described earlier after 30 min.

To confirm ACPH activity in PAM212 cells, we tested the ACPH activity of cell lysates using AcApNA as a substrate (Scaloni *et al*, 1992b, 1994; Sharma and Ortwerth, 1993; Yamaguchi *et al*, 1999) and the influence of inhibitors. Acylpeptide hydrolase has earlier been reported to be inactivated with the thiol reagent pHMB (Scaloni *et al*, 1994) and histidine-modifying reagent DEP (Scaloni *et al*, 1992b). As shown in Figure 7A and B, the PAM212 cell lysate showed some ACPH activity in the absence of inhibitors. However, the ACPH activity was rapidly and completely inhibited by pHMB (Figure 7A) and moderately by DEP (Figure 7B).

To assess if our peptide prodrugs are potential substrates for ACPH, we studied the influence of the concentration of the drug on AcApNA hydrolysis (Figure 7C). We evaluated the influence of ALA, phenylalanyl derivative **1** (the most efficient of the peptide derivatives) and Ac-Ala-Ala-Ala-Ala-OH, which is a substrate for ACPH (Krishna and Wold, 1992; Sokolik *et al*, 1994). 5-Aminolaevulinic acid exerted no significant influence on ACPH activity, whatever the concentration used. The ACPH substrate, Ac-Ala-Ala-Ala-Ala-OH, reduced the ACPH activity by 42 and 47%, respectively, with 5 and 10 mM. Compound **1** exhibited similar effects by a reduction of activity by 25 and 41% with 5 and 10 mM. However, for lower concentrations tested, the activity was reduced by 22 and 28%, respectively, with 0.5 and 1 mM with **1**; no reduction of the activity was observed with Ac-Ala-Ala-Ala-Ala-OH at these concentrations, showing that **1** has a higher affinity for ACPH compared with Ac-Ala-Ala-Ala-Ala-OH.

### Knockdown of ACPH protein expression with siRNA

**Figure 8:**
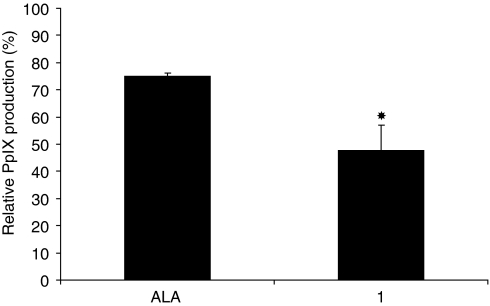
Relative PpIX production (%) in PAM212 cells treated with siRNA directed towards ACPH mRNA. Seventy-two hours after transfection, cells were incubated with 0.5 mM of ALA or **1** during 4 h at 37°C. Error bars show standard deviations. ^*^P<0.01 (*vs* no transfected cells).

To further define the role of ACPH in the ALA peptide derivative hydrolysis, RNA interference for ACPH was employed by transfection of cells with the specific siRNA against murine ACPH mRNA (Figure 8). Inhibition of the ACPH protein expression by siRNA reduced the PpIX production significantly by 53% after incubation with compound **1**. However, when cells were incubated with ALA, the PpIX production was only reduced by 25%. These results convincingly indicate that the expression of ACPH protein is involved in the hydrolysis of ALA peptide derivatives.

## Discussion

The targeted delivery of anticancer drugs to specific cell types, including photosensitisers in PDT, is of crucial importance for minimising treatment side effects. For the ALA prodrugs to exert a pharmacological effect, the derivatives must be able to reach the target sites at a sufficient concentration; this depends on the presence on the target cells of the necessary transport systems able to deliver them into the cytoplasm (Doring *et al*, 1998; Langer *et al*, 1999; Novotny *et al*, 2000; Rud *et al*, 2000; Irie *et al*, 2001) and their ability to undergo an efficient enzymatic hydrolysis to liberate ALA for the subsequent conversion to protoporphyrin IX (Peng *et al*, 1996).

A major challenge in ALA-PDT at the present time is the need to achieve a more effective penetration of ALA into target tissues or the cells of pathogenic organisms. A limitation to the use of ALA in PDT results from its low lipid solubility, a consequence of its zwitterionic character at physiological pH that leads to poor penetration through biological barriers such as cellular membranes. This problem has partly been addressed through the synthesis of lipophilic ester prodrugs that provide improved cellular uptake and are metabolised into PpIX following the action of nonspecific intracellular esterases (Lopez *et al*, 2004). A more recent development concerns the preparation of peptide-based ALA prodrugs, and we (Casas *et al*, 1999; Rogers *et al*, 2005) and others (Berger *et al*, 2000, 2003) have described the synthesis and evaluation of short ALA peptide derivatives in which either the amino or the carboxyl functions of the latter are masked, thereby providing improved physical properties and the potential for cell line-specific ALA release, according to which peptidases are expressed. We have shown earlier the feasibility of using L-phenylalanyl-ALA derivatives (Bourré *et al*, 2008) to enhance intracellular porphyrin production and improve the efficacy of photodynamic treatment.

In this study, we investigated the properties of a larger number of ALA peptide derivatives containing different amino acids and studied the influence of structural modification in the form of the presence or absence of an N-terminal blocking group, compared with ALA itself (Figure 1). The presence of the *N*-acetyl group at the N termini of compounds **1**–**7** prevents decomposition of the derivative at physiological pH. Consequently, these peptide derivatives do not need to be prepared and then dissolved as hydrochloride salts, unlike ALA. We have also prepared the corresponding non-acetylated derivatives, **8** (L-Phe-ALA-Me) and the free acid **9** (L-Phe-ALA). However, these compounds show limited stability, presumably due to the cyclisation of the N-terminal amino group of phenylalanine onto the carbonyl moiety of the ALA residue, generating a six-membered Schiff base intermediate that may react further (Berger *et al*, 2003), which therefore necessitates preparation just before the experiment.

The determination of protoporphyrin IX production (Figure 2) showed that compounds **1**–**6** were found to be capable of being taken up by cells and releasing ALA for subsequent conversion to PpIX, with a significant enhancement of PpIX production compared with ALA for compounds **1**, **6**, **5**, **4** and **3**, respectively.

The effect of modification of the prodrug structure at the C or N terminus on PpIX production, compared with ALA, was then studied. Fluorescence spectroscopy was employed to detect and quantify the PpIX accumulation induced by the compounds **1** and **7**–**10** *vs* ALA in the transformed keratinocyte PAM212 cell line. Compound **7** (Figure 3), corresponding to the free acid of compound **1**, showed a significant increase compared with ALA, with a PpIX level comparable with compound **1**. However, **8** and **9**, compounds without an acetyl group at the N terminus, induced less PpIX compared with ALA. The acetyl group at the N terminus, therefore, seems to play an important role, in metabolic processes leading to the production of PpIX, whereas masking the C terminus as a methyl ester does not exert a major effect. In agreement with the fluorescence pharmacokinetic data, photodynamic treatment of cells incubated with ALA, **1**, **6**, **7**, **8** and **9** (Figure 5) showed that the photocytotoxicity correlated with the PpIX fluorescence level described in Figure 3A. Following the illumination of cells incubated with compounds **1**, **6** and **7**, cell survival was significantly reduced (Figure 5) compared with equimolar ALA. However, ALA, **8** and **9** exhibited no phototoxicity in agreement with the lack of porphyrin produced by these compounds.

Substitution (compound **10**) on the N terminus by a benzyloxycarbonyl (Z) group, instead of the acetyl group, abolished the activity of the compound with no PpIX production observed, as shown in Figure 4A. In pig skin explants, we have shown earlier (Bourré *et al*, 2008) that **1** was able to induce higher porphyrin fluorescence by a factor 3 compared with ALA at 0.2 mM drug concentration; however, once again no porphyrin production was found with the Z derivative (data not shown). Finally, the presence of the amino acid in the derivative was studied, and for this purpose the molecule, Ac-ALA-Me, was synthesised (Figure 4B). No PpIX production was observed with Ac-ALA-Me compared with ALA or **1**, whatever the incubation time or the concentration used. In conclusion, the nature of the N terminus appears to play an important role in the efficacy of these compounds, which indicates that the release of the substituted amino acid intracellularly is probably conditioned by a specific enzymatic activity.

Berger *et al* (2000) have described the evaluation of several L-amino-acid ALA derivatives and their PpIX production in different cell lines (rat endothelial EC212, human endothelial HCEC and lung carcinoma A549 cells). Included in this study was compound **8**, which showed high yields of PpIX in all cell lines compared with other neutral and charged amino-acid derivatives investigated. We have also observed PpIX production with this derivative in the PAM212 cell line, although it is far less efficient than the corresponding N-terminally acetylated derivative **1,** which is moreover far more stable at physiological pH. Berger *et al* (2000) showed the involvement of aminopeptidase N/M activity in ALA release from **8** and other prodrugs in various cell lines. These enzymes, which are located both at the cell surface and in the cytoplasm, show a strong preference for substrates that contain neutral amino-acid residues at the N terminus. In agreement with our findings here, Berger *et al* (2000) observed no PpIX production in cells from peptide derivatives in which the N terminus was blocked with an alkoxycarbonyl group (*t*-butoxycarbonyl, Boc). Such derivatives are not expected to be substrates for aminopeptidase N/M. Uncapped compounds **8** and **9**, which are potential substrates for aminopeptidase N/M, showed low PpIX production. We did, however, find that PAM212 cells had APN/M activity using the specific substrates H-Ala-*β*NA, H-Phe-*β*NA and H-Leu-*β*NA (data not shown), suggesting that the lack of PpIX production with **8** and **9** may be due to poor cellular uptake.

We also studied the influence of the aminopeptidase inhibitors (amastatin, bestatin and aminopeptidase N inhibitor) on PpIX production with **1** (data not shown), but none of them were able to reduce the PpIX production, confirming that the *N*-acetyl ALA peptide derivatives studied here should not be substrates for aminopeptidases and that these enzymes are not involved in the mechanism of ALA release from these ALA peptide derivatives. It is more probable that endopeptidases or exopeptidases, which can act on N-terminally acylated derivatives, are involved. On the basis of the results described here, it is probable that the *N*-acetylated amino-acid residue is cleaved to release ALA or ALA-Me for conversion to PpIX. We hypothesised that the acylpeptidyl residue is most likely released by the acylpeptide hydrolase (ACPH, EC 3.4.19.1), a member of the prolyl oligopeptidase family of serine peptidases, and an exopeptidase that removes acylated amino-acid residues from the N terminus of oligopeptides.

To obtain further information concerning the enzymatic activities involved in releasing free ALA from these precursors, we evaluated PpIX production in the presence of class-specific protease inhibitors (Figure 6): protease inhibitor cocktail, aprotinin, AEBSF, 2,2′-bipyridyl, bestatin, E64, leupeptin and captopril. The PpIX production was reduced by the serine protease inhibitors, aprotinin and AEBSF.

The ACPH activity of the PAM212 cellular extract was then assessed using pAcNA as a substrate (Scaloni *et al*, 1992b, 1994; Sharma and Ortwerth, 1993; Yamaguchi *et al*, 1999). The PAM212 cell lysate showed some ACPH activity in the absence of inhibitors. However, the ACPH activity was rapidly and completely inhibited by pHMB (Figure 7A) and moderately by DEP (Figure 7B). The influence of preincubation of cell extracts with ALA peptide derivative on the ACPH activity was studied, showing that compound **1** induced a reduction of the activity by 41% at 10 mM. These results were similar to those obtained with Ac-Ala-Ala-Ala-Ala-OH, an ACPH substrate, and are therefore consistent with the involvement of the serine protease AC0PH in ALA release from ALA-containing peptide derivatives.

The final functional argument to prove the causal relationship between ACPH expression/activity and ALA peptide derivative hydrolysis inducing the PpIX formation is now provided by the fact that the PpIX formation can be reduced by siRNA interference directed towards ACPH (Figure 8). Sequence-specific post-transcriptional gene silencing by double-stranded RNA (dsRNA), also called RNA interference, is conserved in a diverse variety of organisms (Gupta *et al*, 2004). RNA interference is mediated by siRNAs that are produced from long dsRNAs of exogenous or endogenous origin by an endonuclease of the ribonuclease-III type. The resulting siRNAs are 21–23 nucleotides long and are incorporated into a nuclease complex. After binding to the RNA-induced silencing complex, complementary mRNA is cleaved (Elbashir *et al*, 2001). Here, we described siRNA sequences effective in reducing ACPH expression on reverse transfection, leading to a significant PpIX reduction by 53% with compound **1**. These results confirm that the expression of ACPH protein is involved in the hydrolysis of the ALA peptide derivatives. Moreover, it is known that the enzyme is preferentially directed towards peptides with Ac-Ala- and Ac-Met-, and tolerates a variety of residues in the second position (P_1′_) (Jones and Manning, 1988; Radhakrishna and Wold, 1989; Krishna and Wold, 1992; Sokolik *et al*, 1994; Raphel *et al*, 1999). This is consistent with the results obtained with compound **6** corresponding to methionyl derivative, which shows a higher PpIX enhancement compared with the other derivatives, apart from **1,** which also induces similar levels of PpIX. Compound **3**, corresponding to the alanyl derivative, induced a PpIX enhancement compared with ALA but not as high as that of the methionyl or phenylalanyl derivative. However, this result does not reflect the ACPH substrate specificity described in the literature (Kobayashi and Smith, 1987; Krishna and Wold, 1992; Sokolik *et al*, 1994), in which it was found that Ac-Ala-Ala-Ala is the substrate that exhibits the highest hydrolysis efficiency. The hydrolysis parameters were generally determined on specific sequences by studying the influence of the position of one amino acid in the sequence on the hydrolysis rate. In the case of the compounds studied here, no corresponding data are available on the ACPH activity towards peptides including 5-ALA, which mimic Gly-Gly dipeptides, that is with a general sequence Ac-aa-Gly-Gly. These discrepancies need to be examined in terms of the sequence in which the presumed specificity-determining amino acids were displayed in the different substrates, and it would be an oversimplification to merely state that ACPH requires a given residue in position P1, P1′, P2′ and so on. One also has to consider how the activity will be interdependent on the presence of these residues in conjunction with all the other constituents of the peptide substrate. A further potentially important factor is that owing to the presence of the phenyl group, the phenylalanyl derivative is more lipophilic than the alanyl derivative, which should enhance the relative cellular uptake of the phenylalanyl derivative and therefore the measured porphyrin level.

In conclusion, the results in this study provide further evidence that the conjugation of ALA with specific amino acids is a promising approach for inducing enhanced intracellular porphyrin production for photodynamic therapy. As peptidase expression is generally different between normal and tumour cells or tissues, there is the possibility of more specific targeting, particularly by modulating the N-terminal portion. Our derivatives showed a specific affinity for ACPH, an enzyme the expression of which is higher in tumour cells compared with normal cells (Schoenberger *et al*, 1986). Indeed, although ACPH activity is practically absent in A549 small-cell lung carcinoma cells (Scaloni *et al*, 1992a), we were still able to observe low levels of PpIX production after incubation with compound **1** (data not shown). The peptide prodrugs described herein may be suitable for both topical use (e.g. for bladder and cervical cancers in addition to basal cell carcinomas) and for systemic administration in view of their low inherent toxicity (Bourré *et al*, 2008) compared with the hexyl ester of ALA. Further studies to optimise peptide-based ALA delivery are currently in progress using more complex ALA-containing amino-acid sequences and cell-penetrating peptide carriers (Dixon *et al*, 2007).
